# Effects of Single Exposure of Sodium Fluoride on Lipid Peroxidation and Antioxidant Enzymes in Salivary Glands of Rats

**DOI:** 10.1155/2013/674593

**Published:** 2013-04-27

**Authors:** Paula Mochidome Yamaguti, Alyne Simões, Emily Ganzerla, Douglas Nesadal Souza, Fernando Neves Nogueira, José Nicolau

**Affiliations:** Departamento de Biomateriais e Biologia Oral, Faculdade de Odontologia, Universidade de São Paulo, 05508-000 São Paulo, SP, Brazil

## Abstract

Many studies suggest that fluoride exposure can inhibit the activity of various enzymes and can generate free radicals, which interfere with antioxidant defence mechanisms in living systems. To further the understanding of this issue, this present study examined the effects of low-dose fluoride treatment on the activity of enzymatic antioxidant superoxide dismutase (SOD) and catalase (CAT), as well as the levels of lipid peroxidation (LPO) in the parotid (PA) and submandibular (SM) salivary glands of rats. Rats were injected with a single dose of sodium fluoride (NaF) (15 mg F^−^/kg b.w.) then euthanized at various time intervals up to 24 hours (h) following exposure. NaF exposure did not cause significant differences in SOD or CAT activity or LPO levels in PA glands compared to control. Conversely, SM glands presented increased SOD activity after 3 h and decreased SOD activity after 1, 12, and 24 h, while LPO was increased after 6, 12, and 24 h of the NaF injection. There were no significant differences in the CAT activity in the groups studied. Our results demonstrated that NaF intoxication caused oxidative stress in salivary glands few hours after administration. These changes were more pronounced in SM than in PA gland.

## 1. Introduction

Fluoride is widely regarded as the cornerstone of modern preventive dentistry. Because of its cariostatic properties, fluoride has been increasingly added to alternative delivery systems, such as toothpastes and mouth rinses, so that exposure of populations to fluoride other than through fluoridated water supplies and foodstuffs has become significant [1]. The widespread use of these fluoridated products, in addition to its ubiquitous presence in the environment, has renewed consideration of the margin which exists between safe and toxic levels of fluoride exposure [2, 3].

Although the most pronounced effects of fluoride intake are manifested in bones and teeth, it is also known to cross cell membranes by simple diffusion and enter soft tissues causing adverse effects on cell metabolism and function [1, 4–6]. In soft tissues, its concentration is proportional to the plasma concentration [7]. Salivary glands are important secretory organs, vital to various processes occurring in the oral cavity. Their secretory products have an utmost importance for several physiological functions, playing a critical role in oral and systemic health by monitoring, regulating, and maintaining the integrity of the oral hard and soft tissues [8]. The major salivary glands of both humans and rodents consist of three pairs of macroscopic glands: parotid (PA), submandibular (SM), and sublingual [9]. 

Studies with low doses of NaF administered to experimental animals have been shown to induce a number of alterations in the metabolism of their salivary glands. Some of these metabolic alterations include increases in glycogen content in SM glands [10] and higher levels of 3′, 5′cyclic AMP (cAMP) in PA and SM glands [11] as well as promoting the release of high molecular weight mucins from the SM gland [12]. Fluoride is also known to inhibit the activity of many enzymes [13, 14]. Some of the effects reported arise indirectly because one pathway is inhibited by fluoride making more substrate available for other pathways which thus appear to be enhanced [14]. NaF in low concentrations can alter activities of some carbohydrate metabolizing enzymes such as phosphofructokinase-1, hexokinase, pyruvate kinase, glucose-6-phosphate dehydrogenase, and lactate dehydrogenase in SM glands of rats [15, 16] and promote the release of amylase secretion from PA glands of rats and humans [12].

Oxidative stress is biomolecular damage caused by the attack of reactive species (RS) upon the constituents of a living organism [17]. Among RS, reactive oxygen species (ROS) play a major part because they are highly reactive and formed by numerous enzymes [18]. The production of excessive amounts of ROS is toxic to the cell. The human body has different methods of reducing the impact of oxidative injury, using enzymatic or nonenzymatic defence systems to prevent oxidative stress damage or by repairing the damage after it has occurred [19]. The antioxidant defence systems such as antioxidant vitamins (vitamins A, C, and E), SOD, CAT, glutathione (GSH), and glutathione peroxidase (GSH-Px) protect the cells against LPO [20].

The problems associated with F exposure is that it amplifies the biochemical stress in the body by generating imbalance between ROS and antioxidants thereby inducing oxidative stress and inhibiting several groups of enzymes [13, 14, 21], including many whose action depends on divalent metals such as magnesium (enolase, phosphatases) or trivalent metals (catalase, peroxidase) [14]. These effects have been observed in several soft tissues and cells, such as brain [21–26], gastrocnemius muscle [26], kidney [21, 23–25, 27–29], liver [19, 21, 23–25, 28, 30–32], heart [21], nervous system [33], blood [5, 25, 28, 34, 35], and osteoblasts [36, 37].

However, to the best of our knowledge, no information exists concerning the relation between fluoride intake and oxidative stress in salivary glands. Therefore, the investigation reported herein was undertaken to evaluate the influence of a low dose of NaF over a time period of 24 h on some antioxidant enzymes and LPO in SM and PA salivary glands of rats.

## 2. Material and Methods

### 2.1. Chemicals and Reagents

Sodium fluoride (NaF) (CAS no. 7681-49-4), nicotinamide adenine dinucleotide phosphate, reduced form (NADPH) disodium salt, 2-mercaptoethanol, ethylene-diamine-tetra-acetic acid (EDTA), triethanolamine, diethanolamine, and trichloroacetic acid were purchased from Sigma Aldrich Co. (St. Louis, MO, USA). 2-Thiobarbituric acid was obtained from Merck KGaA (Darmstadt, Germany). All other chemical reagents were of highest pure analytical grade commercially available.

### 2.2. Ethical Aspects of Research

Experimental protocols and animal handling and care were conducted in compliance with the guidelines established by the Brazilian College of Animal Care (COBEA) and according to the standards of humane treatment for animals. This study was approved by the Bioethics Committee of Animals from School of Dentistry, University of São Paulo, approval number 01/06.

### 2.3. Experimental Design and Sample Preparation

One hundred two-month-old male rats of Wistar strain were used in the present investigation. Animals were obtained from the Department of Biomaterials and Oral Biochemistry, School of Dentistry (FOUSP), University of São Paulo, São Paulo, Brazil.

Rats were housed in solid bottomed polypropylene cages, acclimatized for 7 days to animal house conditions, and maintained locally with *ad libitum* commercially available rodent chow diet (Purina) and tap water. The fluoride concentration in the tap water of São Paulo is regulated by the city government at 0.7 ppm. Drugs were freshly prepared prior to administration. Prior to the treatment, all rats' body weights (b.w.) were obtained to minimize intergroup differences. Animals weighed between 220–270 g and, therefore, were randomly and equally (*n* = 50) stratified into two groups according to the treatment received, fluoride (F) and control (C). Fluoride treatment groups were intraperitoneally administered with a single injection of NaF solution (15 mg F^−^/kg b.w.), and control rats received an equivalent dose of sodium chloride solution (0.9% NaCl). Each treatment group, F and C, was further divided into 5 subgroups according to the length of time after injection. The animals were euthanized 1, 3, 6, 12, and 24 h after injection, and SM and PA salivary glands were immediately excised, cleaned in isotonic solution, precooled in dry ice, and stored at −80°C until further processing. Tissues were minced and homogenized in a T-8 Ultra-Turrax homogenizer (IKA-Werke GMBH & CO.KG, Germany) at 10% (w/v) in an ice-cold 50 mM phosphate buffer solution (PBS), pH 7.0. To remove red blood cells, tissue samples were washed twice with 5 volumes of 0.9% NaCl solution. Fibrous material and other tissue debris were eliminated by centrifugation of the tissue homogenate at 1,540 ×g for 10 min at 4°C (Himac CF 15R, Hitachi, Japan), and the supernatants were used for all determinations.

### 2.4. Assay Procedures

All assays were monitored at 25°C in a model DU-800 spectrophotometer (Beckman, Fullerton, CA, USA).

Specific activity of CAT (EC 1.11.1.6) was determined by following the decomposition of hydrogen peroxide (H_2_O_2_) at 240 nm for 3 min and calculated using the molar extinction coefficient of 43.6 M cm^−1^. One unit of activity is defined as the amount of the enzyme required to decompose 1 *μ*mol of H_2_O_2_/min [38, 39]. 

Specific activity of total SOD (EC 1.15.1.1.) was determined measuring inhibition of superoxide-driven NADPH oxidation by mercaptoethanol in the presence of EDTA and manganese (II) chloride. Changes in the absorbance were measured at 340 nm. Percent inhibition was used as the index of SOD activity and calculated as (sample rate)/(control rate) × 100; one unit of SOD activity was defined as half-maximal inhibition [40, 41].

Malondialdehyde, the marker of extent lipid peroxidation, was estimated as thiobarbituric acid reactive substances (TBARS) level in gland tissue by the method of Esterbauer and Cheeseman [42]. Samples were read at 532 nm, and the amount of TBARS was calculated using a molar extinction coefficient of 1.56 × 10^5^ M/cm.

Protein content was determined by using Folin-phenol reagent with bovine serum albumin as standard by the method of Lowry et al. [43].

### 2.5. Statistical Analysis

All experiments were performed in duplicates, and the values are expressed as mean ± standard deviation (SD). All data were checked for normality and analyzed by one- (factor: treatment) or two-way (factors: treatment and time) analysis of variance (ANOVA). When significant main effects were detected in the outcome measures of the study (treatment × time), the means were subsequently analyzed by Tukey test for all pairwise comparisons. All statistical tests were performed using Minitab Statistical Software (PA, USA). Differences were considered statistically significant at *P* < 0.05.

## 3. Results

### 3.1. Effects of NaF on LPO of PA and SM Glands

**Figure 1:**
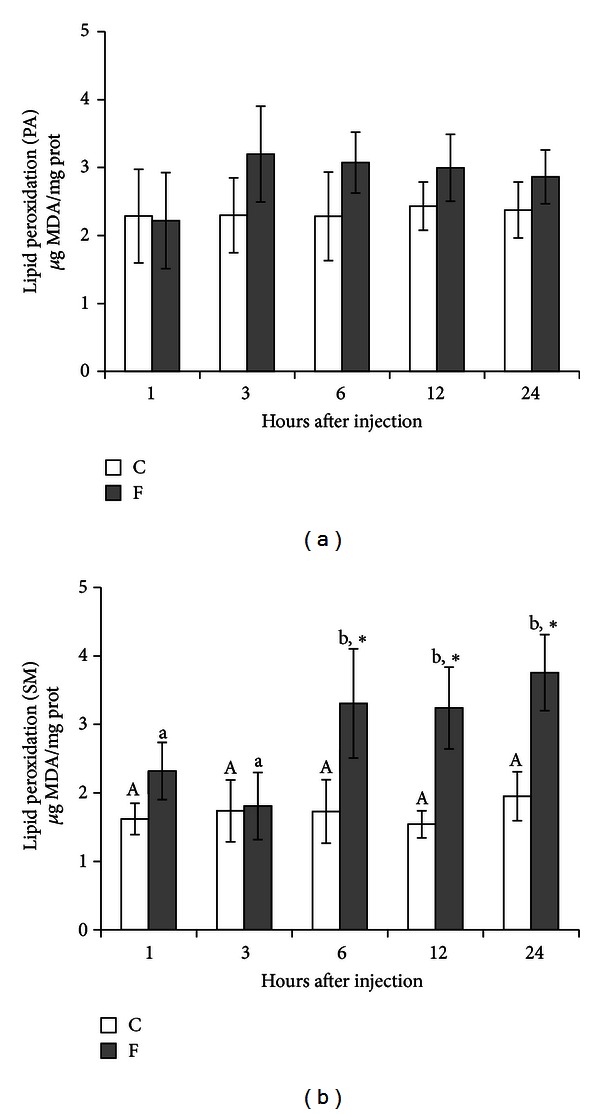
Levels of MDA in PA (*n* = 6) and SM (*n* = 10) glands of rats after treatment with a single IP injection of 15 mg F^−^/kg b.w. in the experimental group (F) and with 0.9% NaCl in the control group (C). Rats were euthanized after 1, 3, 6, 12, and 24 h. No difference was observed in PA. *P < 0.05 compared to the control group. Different letters show *P* < 0.05 for different time intervals in the same treatment group.

Figure 1 shows the levels of MDA in PA (*n* = 6) and SM (*n* = 10) glands of rats, respectively, after injection of 15 mg F^−^/kg b.w. Though the levels of MDA in PA glands were marginally higher in experimental groups (F), they were not statistically significant. In the SM glands, animals treated with fluoride presented higher levels of MDA production than the control group. The values were 91%, 110%, and 93% after 6, 12, and 24 h of the NaF injection, respectively (*P* < 0.01).

### 3.2. Specific Activity of CAT in PA and SM Glands after Treatment with Fluoride

**Figure 2:**
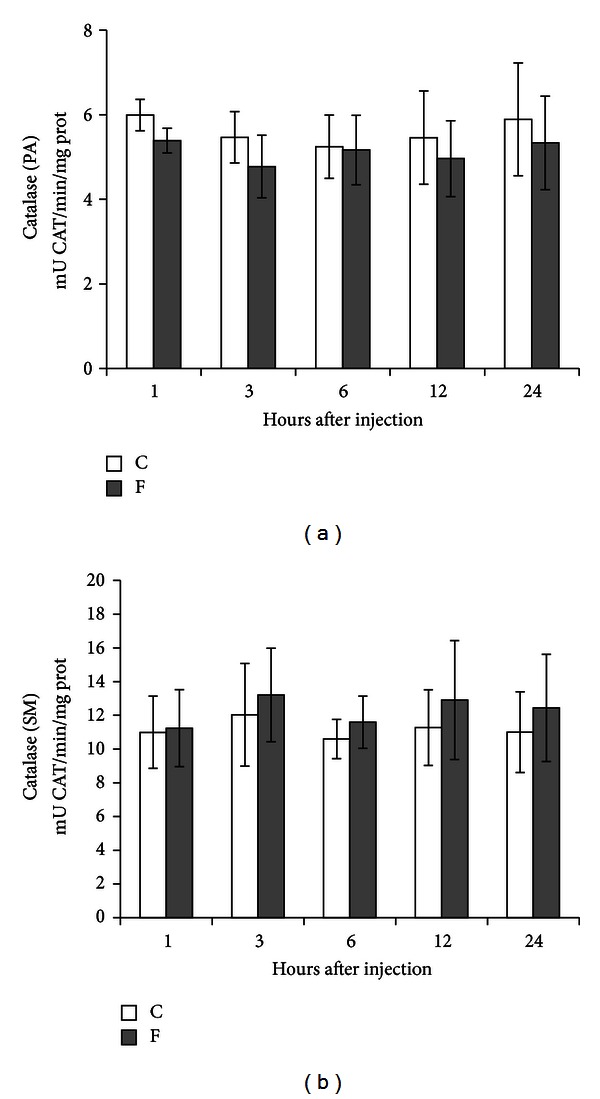
Specific activity of CAT in PA (*n* = 6) and SM (*n* = 10) glands of rats after treatment with a single IP injection of 15 mg F^−^/kg b.w. in the experimental group (F) and with 0.9% NaCl in the control group (C). Rats were euthanized after 1, 3, 6, 12, and 24 h. No difference was observed in PA and SM.

Figure 2 presents the specific activity of CAT in PA (*n* = 6) and SM (*n* = 10) glands of rats, respectively, after injection of 15 mg F^−^/kg b.w. Although, fluoride exposure promoted a very slight decrease in CAT activity of PA glands, no significant differences were observed in any time point within the studied groups. We observed a discrete increase trend in the activity of CAT in SM glands; however, it was also not significant (*P* = 0.056).

### 3.3. Total Activity of SOD in PA and SM Glands after Treatment with Fluoride

**Figure 3:**
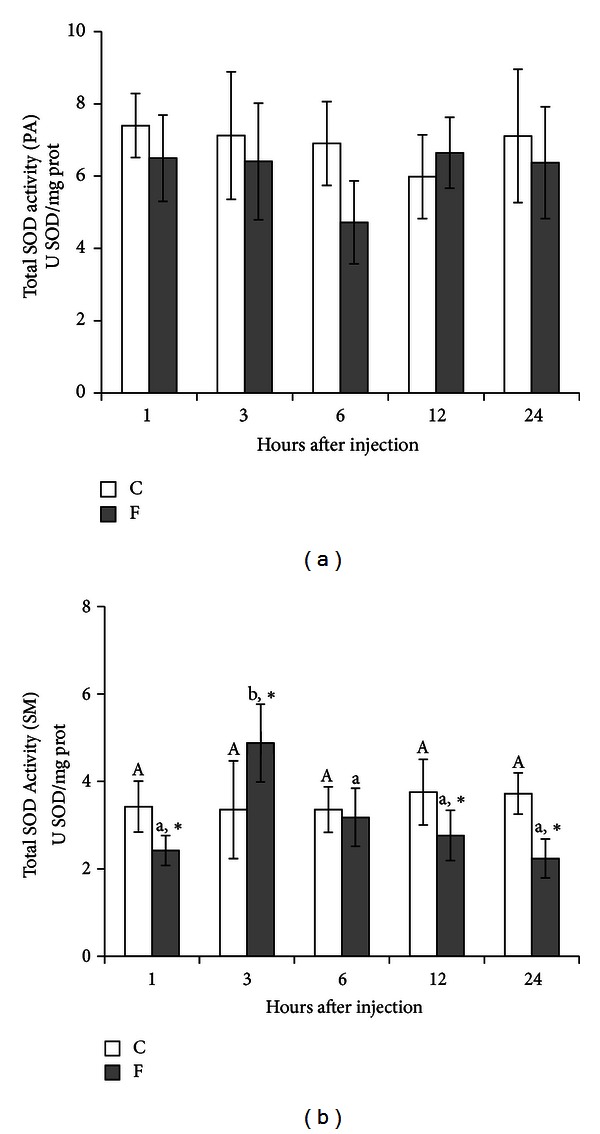
Total activity of SOD in PA (*n* = 6) and SM (*n* = 10) glands of rats after treatment with a single IP injection of 15 mg F^−^/kg b.w. in the experimental group (F) and with 0.9% NaCl in the control group (C). Rats were euthanized after 1, 3, 6, 12, and 24 h. No difference was observed in PA. *P < 0.05 compared to the control group. Different letters show *P* < 0.05 for different time intervals in the same treatment group.

Figure 3 shows the total activity of SOD in PA (*n* = 6) and SM (*n* = 10) glands of rats, respectively, after injection of 15 mg F^−^/kg b.w. Once again, fluoride treatment did not aggravate the activity of SOD in PA glands. As for SM glands, fluoride induced a different response. SM glands showed a significant reduction of SOD activity after 1 h (29%), 12 h (26%), and 24 h (40%) of NaF administration (*P* < 0.05). Conversely, a significantly increased activity of SOD (46%) (*P* < 0.05) was observed after 3 h.

## 4. Discussion

This study assessed the susceptibility of SM and PA salivary glands to oxidative stress and LPO induced by a single injection of a low concentration of fluoride over a period of 24 h. Injections of NaF (15 mg F^−^/kg b.w.) resulted in very slight alterations in PA salivary glands of rats. SM glands presented an increase in SOD activity after 3 h and a decrease of its activity after 1, 12, and 24 h, while LPO was substantially increased after 6, 12, and 24 h. Another relevant observation in our study is that the time period after NaF injection did not influence PA gland response as it did on SM gland. 

The mechanism by which fluoride produces its effects has still not been elucidated, and therefore, the manner in which whole body effects are produced is still unclear [4, 44, 45]. Many studies have proposed that fluoride in varying concentrations induces increased ROS generation, enhanced LPO, and impaired antioxidant enzyme defence system in blood and tissues of experimental animals by interfering with the major metabolic pathways of the living system [26, 28, 45–47].

Antioxidant protection of living organism consists of several levels of defensive response activity including enzymes, proteins, and low-molecular-mass agents [47]. The mitochondrial electron transport chain and a variety of cellular oxidases are the main source of ROS, which are continuously generated as by-products of various intracellular redox reactions. The primary defences against oxidative injury are the antioxidant enzymes that control RS metabolism. One detoxifying enzyme that counteracts potentially deleterious-oxidizing agents is superoxide dismutase. SOD converts the dismutation of superoxide anions (O_2_^•−^) to a less reactive nonradical specie, H_2_O_2_, in the presence of metal ions (copper and iron). It represents the primary line of ROS defence, as it prevents further generation of free radicals, by being highly efficient in catalytic removal of O_2_^•−^ [17]. Published scientific literature reports that fluoride in varying concentrations impaired SOD activity in liver, kidney, brain, thyroid, and cultured cells [19, 26, 27, 29, 44, 45, 48], increased activity in osteoblasts [49] and effected no change in red blood cells [5]. In the SM gland particularly, treatment with fluoride decreased SOD activity after 1, 12, and 24 h and an increase after 3 h (Figure 3). This increase in SOD activity after 3 h of NaF administration may suggest an adaptive and transient response to fluoride intoxication [21]. The loss of SOD activity may be explained due to the fact that fluoride ions are among competitive inhibitors of SOD activity and the reaction rate for fluoride binding to the active site reaches an equilibrium within a very short period of time [50]. Alternately, it could be attributed to a direct action by fluoride on the enzyme leading to the diminished ability of the tissues to handle O_2_^•−^ radicals [51].

CAT subsequently reduces the H_2_O_2_ produced by SOD to water. Catalase is a hemeprotein, which catalyses a dismutation reaction; one H_2_O_2_ is reduced to H_2_O, and the other oxidized to ground-state O_2_ [17]. Authors investigating the influence of fluoride on the activity of CAT reported contradictory results. Some studies have reported decreased [19, 22, 26–29], increased [21], and unchanged [45] CAT activity. Reddy et al. did not find any difference in the activity of CAT in red blood cells of fluoride-intoxicated rabbits [5]. In this investigation, although no changes were observed for the activity of CAT in both PA and SM glands over the studied intervals after fluoride treatment, we did observe a trend towards enhanced activity in SM glands of the experimental group (statistically insignificant, *P* = 0.056).

ROS react with antioxidants and attack redox-sensitive biomolecules. Reactions with these targets result in the cell damage frequently associated with oxidative stress [18]. They react with methylene groups of polyunsaturated fatty acids, initiating the peroxidation of membrane lipids and producing MDA as one of the end products [47]. MDA is considered to be the most significant indicator of membrane LPO arising from the interaction of reactive oxygen types with cellular membranes. Increased LPO from fluoride toxicity may be due to the generation of ROS by high levels of H_2_O_2_ being formed in cells by controlled pathways. H_2_O_2_ at high concentration is deleterious to cells, and its accumulation causes oxidation of cellular targets such as proteins, lipids, and DNA leading to mutagenesis and cell death [52]. Removal of H_2_O_2_ from cells is, therefore, necessary for protection against oxidative damage. In this study, the exposure to a low concentration of fluoride altered the MDA content in SM salivary glands. A marked increase in the concentration of MDA was observed in SM glands of the experimental animals after 6, 12, and 24 h. These data corroborate with many authors who have observed increased levels of MDA in different tissues and cells of fluoride-intoxicated animal [19, 21–24, 27–29, 31, 44, 45, 48, 53].

Extensive amounts of available information concerning the role of fluoride in oxidative stress are inconclusive and conflicting. Reddy et al. suggested that oxidative stress may not be directly related to fluoride toxicity but could be a secondary effect [5]. On the other hand, it is relevant to state that many other factors could have influenced the outcome response to fluoride exposure among these studies, such as diet, route of administration, gender, species, body weight and age of experimental animals, acid-base status, and fluoride compound [13]. Rats are more resistant to fluoride than sheep and rabbits [4]. Younger rats of both sexes are more resistant than older rats, with females being less resistant than males of the same age [14]. In our investigation, the dose of 15 mg F^−^/kg b.w. is relatively low and corresponds to approximately 1/6 of the 24 hour median lethal dose (LD_50_) for a rat intraperitoneally injected with NaF, which has been reported to range from 85.5 to 98.0 mg F^−^/kg [13, 54]. Small amounts of fluoride have been shown to cause normal plasma fluoride levels to surge and peak to potentially harmful values [3]. It has been demonstrated that NaF at a concentration as low as 0.5 mg F^−^/kg increases cAMP levels in PA gland of rats, altering salivary function [12]. IP injections of NaF (15 mg F^−^/kg b.w) increased cAMP concentration in SM gland of rats after 30 and 60 min [55]. The same increased level of cAMP was observed for PA gland cells of rats incubated 0.01 mmol/L of NaF after 10 min [56]. Low concentrations stimulate LPO, and at high and very high concentrations may act as inhibitor of MDA generation [47]. Xu et al. reported that low concentrations of fluoride increased activity of antioxidant enzymes and enhanced LPO in osteoblasts of mice [49]. Moreover, the different responses found between SM and PA salivary glands could be possible due to metabolic differences between the two glands: PA gland metabolism is predominantly aerobic, and SM gland metabolism is predominantly anaerobic [57] in addition to distinct histological characteristics and secretion end products in each [58]. Nagler et al. reported that PA saliva secretion presents much higher levels of salivary molecular and enzymatic antioxidants parameters than SM/SL saliva [59], which corroborate with our findings, where PA salivary glands were more able to cope with oxidative stress induced by NaF exposure than SM gland.

In conclusion, the results observed in this present study have demonstrated that intraperitoneal administration of a low concentration dose of NaF caused impairments in the antioxidant defence system in the salivary glands of experimental animals. Specifically, SOD activity was decreased while LPO was increased in the first hours after intoxication. Also significant was that the oxidative stress induced by NaF intoxication was more pronounced in the SM gland than in the PA gland.
